# *trans*-4-Nitro­phenyl 4-(tosyl­oxymeth­yl)cyclo­hexa­necarboxyl­ate

**DOI:** 10.1107/S1600536808000068

**Published:** 2008-01-09

**Authors:** Qing-Rong Qi, Wen-Cai Huang, Zhi-Hua Mao, Hu Zheng

**Affiliations:** a Department of Medicinal Chemistry, West China School of Pharmacy, Sichuan University, Chengdu 610041, People’s Republic of China; b Department of Pharmaceutical and Bioengineering, School of Chemical Engineering, Sichuan University, Chengdu 610065, People’s Republic of China; c The Center for Testing and Analysis, Sichuan University, Chengdu 610064, People’s Republic of China

## Abstract

The title compound, C_21_H_23_NO_7_S, is an important inter­mediate in the synthesis of poly(amido­amine) dendrimers. The cyclo­hexane ring adopts a chair conformation. The dihedral angle between the two aromatic rings is 69.5 (2)°. The mol­ecules are linked into a zigzag chain along the *b* axis by C—H⋯O hydrogen bonds.

## Related literature

For related literature, see: Bucourt & Hainaut (1965 ▶); Dunitz & Strickler (1966 ▶); Sewald & Jakubke (2002 ▶); Luger *et al.* (1972 ▶); van Koningsveld & Jansen (1984 ▶).
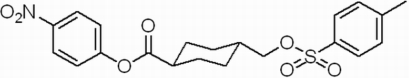


## Experimental

### 

#### Crystal data

C_21_H_23_NO_7_S
*M* *_r_* = 433.46
Monoclinic, 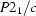

*a* = 20.499 (5) Å
*b* = 6.111 (3) Å
*c* = 17.250 (4) Å
β = 102.04 (3)°
*V* = 2113.4 (13) Å^3^
*Z* = 4
Mo *K*α radiation
μ = 0.20 mm^−1^
*T* = 292 (2) K
0.40 × 0.38 × 0.30 mm

#### Data collection

Enraf–Nonius CAD-4 diffractometer
Absorption correction: none
4094 measured reflections
3627 independent reflections
1850 reflections with *I* > 2σ(*I*)
*R*_int_ = 0.004
3 standard reflections every 300 reflections intensity decay: 2.1%

#### Refinement

*R*[*F* ^2^ > 2σ(*F* ^2^)] = 0.060
*wR*(*F* ^2^) = 0.191
*S* = 1.06
3627 reflections
272 parameters
H-atom parameters constrained
Δρ_max_ = 0.40 e Å^−3^
Δρ_min_ = −0.53 e Å^−3^

### 

Data collection: *DIFRAC* (Gabe & White, 1993 ▶); cell refinement: *DIFRAC*; data reduction: *NRCVAX* (Gabe *et al.*, 1989 ▶); program(s) used to solve structure: *SHELXS97* (Sheldrick, 2008 ▶); program(s) used to refine structure: *SHELXL97* (Sheldrick, 2008 ▶); molecular graphics: *ORTEP-3 for Windows* (Farrugia, 1997 ▶); software used to prepare material for publication: *SHELXL97*.

## Supplementary Material

Crystal structure: contains datablocks global, I. DOI: 10.1107/S1600536808000068/ci2549sup1.cif

Structure factors: contains datablocks I. DOI: 10.1107/S1600536808000068/ci2549Isup2.hkl

Additional supplementary materials:  crystallographic information; 3D view; checkCIF report

## Figures and Tables

**Table 1:** Hydrogen-bond geometry (Å, °)

*D*—H⋯*A*	*D*—H	H⋯*A*	*D*⋯*A*	*D*—H⋯*A*
C5—H5⋯O7^i^	0.93	2.49	3.290 (6)	144

Symmetry code: (i) 
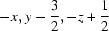
.

**Fig. 1.**
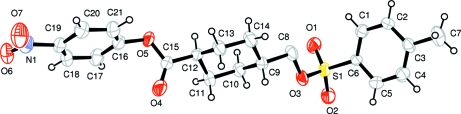
The molecular structure of (I), showing the atomic numbering scheme. Displacement ellipsoids drawn at the 30% probability level.

## supplementary crystallographic information

### Comment

Activated esters are commonly used in peptide synthesis to facilitate the reaction of carboxylic acid and amine to obtain amide. *trans*-Nitrophenyl ester of N-protected amino acid can react with amino group quickly at room temperature with fairly good yields (Sewald *et al.*,2002). In our work, cyclohexane derivatives were designed to be linked to poly(amidoamine)dendrimer[PAMAM] through the amide bond which could be formed by the reaction of terminal amino groups of PAMAM dendrimer and cyclohexanecarboxylic acid. So the title compound, a *p*-nitrophenyl ester of *trans*-4-(tosyloxymethyl) cyclohexanecarboxylic acid was synthesized. As expected, the modification of periphery of PAMAM dendrimer effectively is realised under mild conditions. We report here the crystal structure of the title compound.

The cyclohexane ring of the title compound adopts a chair conformation. The average C—C bond length of the cyclohexane ring is 1.521 (5) Å, which is close to that of *trans*-1,4-cyclohexanedicarboxylic acid (1.523 (3) Å, Von Luger *et al.*, 1972). The mean endocyclic angle of the cyclohexane is 111.08 (3)°, which is close to that observed for cyclohexane rings (111.1°, Bucourt & Hainaut, 1965; 111.4 (4)°, Dunitz & Strickler, 1966; Von Luger *et al.*, 1972).

The molecules are linked into a zigzag chain along the *b* axis by C—H···O hydrogen bonds (Table 1).

### Experimental

*trans*-4-(Tosyloxymethyl)cyclohexanecarboxylic acid (10 mmol) and *p*-nitrophenol (11 mmol) were dissolved in ethyl acetate (10 ml) and the solution was cooled to 273 K in an ice bath. Then a solution of DCC (11 mmol) in ethyl acetate (5 ml) was added dropwsie with stirring. After the addition, the stirring was continued for 30 min at 273 K and for 24 h at room temperature. Then acetic acid (0.5 ml) was added and after 30 min the reaction solution was filtered and the filtrate was evaporated to leave a yellow solid. The title compound was recrystallized from acetone. Single crystals suitable for X-ray analysis were obtained by slow evaporation of a acetone-water solution at room temperature.

### Refinement

H atoms were positioned geometrically (C—H = 0.93–0.98 Å) and refined using a riding model, with *U*_iso_(H) = 1.2–1.5*U*_eq_(C).

### Crystal data

**Table:**

C_21_H_23_NO_7_S	*F*_000_ = 912
*M**_r_* = 433.46	*D*_x_ = 1.362 Mg m^−^^3^
Monoclinic, *P*2_1_/*c*	Mo *K*α radiation λ = 0.71073 Å
Hall symbol: -P 2ybc	Cell parameters from 33 reflections
*a* = 20.499 (5) Å	θ = 4.5–7.6º
*b* = 6.111 (3) Å	µ = 0.20 mm^−^^1^
*c* = 17.250 (4) Å	*T* = 292 (2) K
β = 102.04 (3)º	Block, colourless
*V* = 2113.4 (13) Å^3^	0.40 × 0.38 × 0.30 mm
*Z* = 4	

### Data collection

**Table:**

Enraf–Nonius CAD-4 diffractometer	*R*_int_ = 0.004
Radiation source: fine-focus sealed tube	θ_max_ = 25.0º
Monochromator: graphite	θ_min_ = 1.0º
*T* = 292(2) K	*h* = −8→24
ω/2θ scans	*k* = −7→0
Absorption correction: none	*l* = −20→19
4094 measured reflections	3 standard reflections
3627 independent reflections	every 300 reflections
1850 reflections with *I* > 2σ(*I*)	intensity decay: 2.1%

### Refinement

**Table:**

Refinement on *F*^2^	Secondary atom site location: difference Fourier map
Least-squares matrix: full	Hydrogen site location: inferred from neighbouring sites
*R*[*F*^2^ > 2σ(*F*^2^)] = 0.060	H-atom parameters constrained
*wR*(*F*^2^) = 0.191	*w* = 1/[σ^2^(*F*_o_^2^) + (0.097*P*)^2^] where *P* = (*F*_o_^2^ + 2*F*_c_^2^)/3
*S* = 1.06	(Δ/σ)_max_ = 0.001
3627 reflections	Δρ_max_ = 0.40 e Å^−^^3^
272 parameters	Δρ_min_ = −0.53 e Å^−^^3^
Primary atom site location: structure-invariant direct methods	Extinction correction: none

### Special details

**Table:**

Geometry. All e.s.d.'s (except the e.s.d. in the dihedral angle between two l.s. planes) are estimated using the full covariance matrix. The cell e.s.d.'s are taken into account individually in the estimation of e.s.d.'s in distances, angles and torsion angles; correlations between e.s.d.'s in cell parameters are only used when they are defined by crystal symmetry. An approximate (isotropic) treatment of cell e.s.d.'s is used for estimating e.s.d.'s involving l.s. planes.
Refinement. Refinement of *F*^2^ against ALL reflections. The weighted *R*-factor *wR* and goodness of fit *S* are based on *F*^2^, conventional *R*-factors *R* are based on *F*, with *F* set to zero for negative *F*^2^. The threshold expression of *F*^2^ > σ(*F*^2^) is used only for calculating *R*-factors(gt) *etc*. and is not relevant to the choice of reflections for refinement. *R*-factors based on *F*^2^ are statistically about twice as large as those based on *F*, and *R*- factors based on ALL data will be even larger.

### Fractional atomic coordinates and isotropic or equivalent isotropic displacement parameters (Å^2^)

**Table:**

	*x*	*y*	*z*	*U*_iso_*/*U*_eq_	
S1	0.30112 (5)	−0.15510 (18)	0.17299 (7)	0.0556 (4)	
O1	0.30040 (14)	−0.0770 (6)	0.09454 (16)	0.0702 (9)	
O2	0.30099 (14)	−0.3830 (5)	0.1871 (2)	0.0763 (10)	
O3	0.23652 (12)	−0.0742 (5)	0.19982 (18)	0.0632 (8)	
O4	−0.09447 (15)	0.2718 (6)	0.1532 (2)	0.0885 (12)	
O5	−0.08914 (13)	0.5252 (5)	0.06303 (18)	0.0686 (9)	
O6	−0.39478 (15)	0.7233 (6)	−0.0126 (2)	0.0813 (11)	
O7	−0.36075 (17)	0.9887 (7)	0.0674 (2)	0.0936 (12)	
N1	−0.35100 (18)	0.8235 (8)	0.0323 (2)	0.0635 (10)	
C1	0.39156 (18)	0.1686 (7)	0.2212 (2)	0.0500 (10)	
H1	0.3740	0.2367	0.1732	0.060*	
C2	0.44258 (19)	0.2665 (7)	0.2753 (2)	0.0536 (11)	
H2	0.4589	0.4016	0.2633	0.064*	
C3	0.46966 (18)	0.1673 (8)	0.3465 (2)	0.0526 (10)	
C4	0.4444 (2)	−0.0304 (9)	0.3636 (3)	0.0650 (13)	
H4	0.4618	−0.0973	0.4119	0.078*	
C5	0.3931 (2)	−0.1337 (8)	0.3100 (2)	0.0593 (12)	
H5	0.3769	−0.2690	0.3220	0.071*	
C6	0.36691 (17)	−0.0320 (6)	0.2393 (2)	0.0440 (9)	
C7	0.5248 (2)	0.2787 (10)	0.4050 (3)	0.0811 (16)	
H7A	0.5125	0.4279	0.4121	0.122*	
H7B	0.5652	0.2753	0.3852	0.122*	
H7C	0.5316	0.2037	0.4550	0.122*	
C8	0.21871 (19)	0.1584 (7)	0.1883 (3)	0.0657 (12)	
H8A	0.2286	0.2104	0.1389	0.079*	
H8B	0.2442	0.2448	0.2313	0.079*	
C9	0.14546 (17)	0.1807 (7)	0.1864 (2)	0.0507 (10)	
H9	0.1370	0.1230	0.2364	0.061*	
C10	0.10207 (18)	0.0551 (7)	0.1192 (2)	0.0527 (10)	
H10A	0.1131	−0.0992	0.1249	0.063*	
H10B	0.1115	0.1046	0.0692	0.063*	
C11	0.02817 (18)	0.0850 (8)	0.1176 (2)	0.0564 (11)	
H11A	0.0178	0.0238	0.1655	0.068*	
H11B	0.0023	0.0067	0.0726	0.068*	
C12	0.00917 (18)	0.3266 (7)	0.1115 (2)	0.0525 (11)	
H12	0.0175	0.3836	0.0614	0.063*	
C13	0.05248 (19)	0.4533 (7)	0.1792 (3)	0.0614 (12)	
H13A	0.0426	0.4050	0.2291	0.074*	
H13B	0.0418	0.6078	0.1732	0.074*	
C14	0.1265 (2)	0.4210 (7)	0.1814 (3)	0.0648 (12)	
H14A	0.1374	0.4839	0.1340	0.078*	
H14B	0.1523	0.4975	0.2269	0.078*	
C15	−0.0628 (2)	0.3620 (8)	0.1128 (2)	0.0545 (11)	
C16	−0.1560 (2)	0.5894 (8)	0.0600 (2)	0.0574 (12)	
C17	−0.2075 (2)	0.4605 (8)	0.0214 (3)	0.0662 (13)	
H17	−0.1994	0.3249	0.0008	0.079*	
C18	−0.2720 (2)	0.5386 (8)	0.0140 (3)	0.0631 (13)	
H18	−0.3080	0.4554	−0.0120	0.076*	
C19	−0.28246 (19)	0.7380 (8)	0.0449 (2)	0.0509 (10)	
C20	−0.2310 (2)	0.8667 (8)	0.0847 (2)	0.0623 (12)	
H20	−0.2392	1.0013	0.1059	0.075*	
C21	−0.1658 (2)	0.7874 (9)	0.0920 (3)	0.0665 (13)	
H21	−0.1297	0.8690	0.1185	0.080*	

### Atomic displacement parameters (Å^2^)

**Table:**

	*U*^11^	*U*^22^	*U*^33^	*U*^12^	*U*^13^	*U*^23^
S1	0.0444 (6)	0.0441 (7)	0.0734 (8)	0.0019 (5)	0.0014 (5)	−0.0017 (6)
O1	0.068 (2)	0.082 (2)	0.0556 (18)	0.0010 (16)	0.0017 (14)	−0.0056 (17)
O2	0.0633 (19)	0.041 (2)	0.114 (3)	−0.0006 (15)	−0.0060 (17)	0.0007 (19)
O3	0.0386 (15)	0.053 (2)	0.096 (2)	0.0042 (13)	0.0114 (14)	0.0135 (17)
O4	0.0562 (19)	0.106 (3)	0.108 (3)	0.0247 (18)	0.0286 (18)	0.052 (2)
O5	0.0447 (16)	0.079 (2)	0.082 (2)	0.0155 (15)	0.0136 (14)	0.0340 (19)
O6	0.0471 (18)	0.101 (3)	0.091 (2)	0.0097 (18)	0.0021 (17)	−0.007 (2)
O7	0.082 (2)	0.105 (3)	0.095 (3)	0.027 (2)	0.0196 (19)	−0.024 (3)
N1	0.060 (2)	0.073 (3)	0.059 (2)	0.018 (2)	0.0138 (19)	0.000 (2)
C1	0.050 (2)	0.047 (3)	0.054 (2)	0.0062 (19)	0.0105 (18)	0.002 (2)
C2	0.052 (2)	0.047 (3)	0.062 (3)	−0.0082 (19)	0.012 (2)	0.003 (2)
C3	0.043 (2)	0.063 (3)	0.054 (2)	−0.004 (2)	0.0146 (18)	−0.001 (2)
C4	0.064 (3)	0.078 (4)	0.050 (2)	−0.004 (2)	0.006 (2)	0.011 (2)
C5	0.061 (3)	0.057 (3)	0.062 (3)	−0.006 (2)	0.016 (2)	0.009 (2)
C6	0.043 (2)	0.034 (2)	0.056 (2)	0.0020 (16)	0.0105 (17)	0.0054 (19)
C7	0.063 (3)	0.106 (5)	0.070 (3)	−0.017 (3)	0.003 (2)	−0.013 (3)
C8	0.043 (2)	0.045 (3)	0.109 (4)	0.0012 (19)	0.015 (2)	0.001 (3)
C9	0.041 (2)	0.047 (3)	0.064 (3)	0.0016 (17)	0.0104 (18)	0.005 (2)
C10	0.050 (2)	0.041 (3)	0.068 (3)	0.0056 (18)	0.0142 (19)	−0.006 (2)
C11	0.047 (2)	0.059 (3)	0.061 (3)	−0.0058 (19)	0.0077 (19)	−0.011 (2)
C12	0.042 (2)	0.063 (3)	0.053 (2)	0.008 (2)	0.0124 (17)	0.010 (2)
C13	0.048 (2)	0.042 (3)	0.095 (3)	0.0063 (19)	0.016 (2)	−0.008 (2)
C14	0.050 (2)	0.046 (3)	0.098 (3)	−0.004 (2)	0.013 (2)	−0.006 (3)
C15	0.050 (2)	0.056 (3)	0.057 (2)	0.006 (2)	0.010 (2)	0.012 (2)
C16	0.047 (2)	0.067 (3)	0.057 (3)	0.008 (2)	0.0088 (19)	0.019 (2)
C17	0.059 (3)	0.056 (3)	0.079 (3)	0.014 (2)	0.005 (2)	−0.014 (3)
C18	0.045 (2)	0.071 (4)	0.067 (3)	−0.001 (2)	−0.001 (2)	−0.004 (3)
C19	0.048 (2)	0.054 (3)	0.049 (2)	0.005 (2)	0.0060 (18)	0.001 (2)
C20	0.062 (3)	0.059 (3)	0.063 (3)	0.007 (2)	0.007 (2)	−0.004 (2)
C21	0.056 (3)	0.069 (4)	0.068 (3)	−0.005 (2)	−0.003 (2)	0.000 (3)

### Geometric parameters (Å, °)

**Table:**

S1—O2	1.414 (3)	C9—C10	1.515 (5)
S1—O1	1.432 (3)	C9—C14	1.517 (6)
S1—O3	1.571 (3)	C9—H9	0.98
S1—C6	1.747 (4)	C10—C11	1.520 (5)
O3—C8	1.471 (5)	C10—H10A	0.97
O4—C15	1.184 (5)	C10—H10B	0.97
O5—C15	1.353 (5)	C11—C12	1.525 (6)
O5—C16	1.416 (5)	C11—H11A	0.97
O6—N1	1.221 (5)	C11—H11B	0.97
O7—N1	1.214 (5)	C12—C15	1.495 (5)
N1—C19	1.472 (5)	C12—C13	1.523 (6)
C1—C2	1.383 (5)	C12—H12	0.98
C1—C6	1.386 (6)	C13—C14	1.523 (5)
C1—H1	0.93	C13—H13A	0.97
C2—C3	1.379 (6)	C13—H13B	0.97
C2—H2	0.93	C14—H14A	0.97
C3—C4	1.371 (6)	C14—H14B	0.97
C3—C7	1.511 (6)	C16—C21	1.362 (6)
C4—C5	1.399 (6)	C16—C17	1.374 (6)
C4—H4	0.93	C17—C18	1.386 (6)
C5—C6	1.373 (5)	C17—H17	0.93
C5—H5	0.93	C18—C19	1.365 (6)
C7—H7A	0.96	C18—H18	0.93
C7—H7B	0.96	C19—C20	1.379 (6)
C7—H7C	0.96	C20—C21	1.402 (6)
C8—C9	1.501 (5)	C20—H20	0.93
C8—H8A	0.97	C21—H21	0.93
C8—H8B	0.97		
			
O2—S1—O1	119.4 (2)	C11—C10—H10A	109.2
O2—S1—O3	103.13 (18)	C9—C10—H10B	109.2
O1—S1—O3	109.29 (18)	C11—C10—H10B	109.2
O2—S1—C6	109.87 (18)	H10A—C10—H10B	107.9
O1—S1—C6	109.28 (19)	C10—C11—C12	110.9 (3)
O3—S1—C6	104.74 (18)	C10—C11—H11A	109.5
C8—O3—S1	117.7 (3)	C12—C11—H11A	109.5
C15—O5—C16	118.9 (3)	C10—C11—H11B	109.5
O7—N1—O6	123.9 (4)	C12—C11—H11B	109.5
O7—N1—C19	118.2 (4)	H11A—C11—H11B	108.0
O6—N1—C19	117.9 (4)	C15—C12—C13	109.5 (3)
C2—C1—C6	119.4 (4)	C15—C12—C11	112.2 (4)
C2—C1—H1	120.3	C13—C12—C11	109.9 (3)
C6—C1—H1	120.3	C15—C12—H12	108.4
C3—C2—C1	121.2 (4)	C13—C12—H12	108.4
C3—C2—H2	119.4	C11—C12—H12	108.4
C1—C2—H2	119.4	C12—C13—C14	111.8 (4)
C4—C3—C2	118.6 (4)	C12—C13—H13A	109.3
C4—C3—C7	121.1 (4)	C14—C13—H13A	109.3
C2—C3—C7	120.2 (4)	C12—C13—H13B	109.3
C3—C4—C5	121.4 (4)	C14—C13—H13B	109.3
C3—C4—H4	119.3	H13A—C13—H13B	107.9
C5—C4—H4	119.3	C9—C14—C13	111.6 (3)
C6—C5—C4	118.9 (4)	C9—C14—H14A	109.3
C6—C5—H5	120.5	C13—C14—H14A	109.3
C4—C5—H5	120.5	C9—C14—H14B	109.3
C5—C6—C1	120.4 (4)	C13—C14—H14B	109.3
C5—C6—S1	119.5 (3)	H14A—C14—H14B	108.0
C1—C6—S1	120.1 (3)	O4—C15—O5	121.4 (4)
C3—C7—H7A	109.5	O4—C15—C12	127.3 (4)
C3—C7—H7B	109.5	O5—C15—C12	111.2 (4)
H7A—C7—H7B	109.5	C21—C16—C17	122.9 (4)
C3—C7—H7C	109.5	C21—C16—O5	117.0 (4)
H7A—C7—H7C	109.5	C17—C16—O5	120.0 (4)
H7B—C7—H7C	109.5	C16—C17—C18	118.0 (5)
O3—C8—C9	108.0 (3)	C16—C17—H17	121.0
O3—C8—H8A	110.1	C18—C17—H17	121.0
C9—C8—H8A	110.1	C19—C18—C17	119.7 (4)
O3—C8—H8B	110.1	C19—C18—H18	120.1
C9—C8—H8B	110.1	C17—C18—H18	120.1
H8A—C8—H8B	108.4	C18—C19—C20	122.6 (4)
C8—C9—C10	113.3 (4)	C18—C19—N1	118.8 (4)
C8—C9—C14	109.4 (3)	C20—C19—N1	118.6 (4)
C10—C9—C14	110.1 (3)	C19—C20—C21	117.6 (5)
C8—C9—H9	107.9	C19—C20—H20	121.2
C10—C9—H9	107.9	C21—C20—H20	121.2
C14—C9—H9	107.9	C16—C21—C20	119.3 (4)
C9—C10—C11	112.2 (3)	C16—C21—H21	120.4
C9—C10—H10A	109.2	C20—C21—H21	120.4

### Hydrogen-bond geometry (Å, °)

**Table:**

*D*—H···*A*	*D*—H	H···*A*	*D*···*A*	*D*—H···*A*
C5—H5···O7^i^	0.93	2.49	3.290 (6)	144

Symmetry codes: (i) −*x*, *y*−3/2, −*z*+1/2.
